# Supporting the Conversational Behavior of Adolescents with Autism Spectrum Disorders with Self-Monitoring and a Video-Based Supplement

**DOI:** 10.1007/s10803-024-06548-3

**Published:** 2024-09-13

**Authors:** Shiri Ayvazo, Yafit Shmuel, Inbar Bin-Nun

**Affiliations:** 1 School of Social Sciences and Humanities, Kinneret Academic College, Galilee, Israel; 2 David Yellin Academic College Jerusalem, 7 Maagal Beit Hamidrash, Jerusalem, Israel

**Keywords:** Autism, Self-monitoring, Video-based model, Conversational behavior

## Abstract

Individuals with autism spectrum disorders (ASD) might demonstrate impairments in initiating and sustaining a conversation and experience conversational challenges such as question-asking and turn-taking. Conversational skills are pivotal for the social functioning of adolescents with ASD. The current investigation aimed to extend the available information on interventions addressing the conversational needs of adolescents with ASD. The research questions were: (a) What is the effect of self-monitoring, supplemented by a video-based model on the conversational skills of adolescent students with ASD? and (b) What is the acceptability of the intervention among the participating adolescents with ASD?. Appropriate conversational behavior of three students with ASD (aged 16–18 years) was assessed using a withdrawal design, during 10-min conversation sessions. Appropriate conversational behavior was defined as a sequence of a turn-taking response (i.e., waiting quietly until the speaker finished talking), followed by a verbal utterance which included (a) making a statement or responding on topic, and/or (b) asking a contextually appropriate “wh”- question. The independent variable consisted of a primary self-monitoring procedure and a daily video-based supplement. Treatment fidelity and treatment acceptability were also assessed. The conversational behavior of all participants consistently improved under the self-monitoring intervention with the video-based supplement. Self-monitoring with a video-based supplement can effectively support the conversational behavior in adolescents with ASD. This information can guide the evaluation and planning of appropriate interventions designed to improve limited conversational behaviors of adolescents with ASD.

**Supplementary Information:**

The online version contains supplementary material available at 10.1007/s10803-024-06548-3.

## Introduction

A core feature of autism spectrum disorders (ASD) is the impairment in social-communication skills including difficulties in initiating and sustaining conversations. Conversational challenges encompass difficulty using social inquiries (i.e., question-asking), conversational fluency, turn taking, perspective taking and initiating and responding while persevering on an appropriate topic of shared interest (Chezan et al., 2020; Detar & Vernon, 2020; Paul et al., 2009; Sng et al., 2018; State & Kern, 2012).

Conversation usually involves exchanging ideas on a shared topic and involves a series of initiations and responses to those initiations while engaging in pragmatic listening and speaking skills (Chezan et al., 2020; Doggett et al., 2013; McKeithan & Sabornie, 2020; Sng et al., 2014). Conversational skills comprise a range of ways to start, end, and respond to social interactions (Bross et al., 2022), discern the listener’s interest in a topic, introduce a topic of shared relevance, ask questions pertinent to the topic, comment clearly with the appropriate amount and type of information the listener needs, and maintain a balanced reciprocity of the conversational exchange – all given changing topics (Doggett et al., 2013; Paul et al., 2009; Sng et al., 2014).

Research supporting conversational skills of individuals with ASD usually focuses on skills of prime concern for sustaining a flowing social conversation such as persevering on a topic, turn taking, asking questions, responding to questions, and commenting on topics (Bross et al., 2022; Doggett et al., 2013; Koegel et al., 1992; Paul et al., 2009; Sng et al., 2018). Given the complex and fluctuating nature of the conversational exchange, conversational skills are often broken down into small discrete responses that can be discretely monitored, prompted, and performed (Sng et al., 2014) with proficiency.

Proficient conversational skills are pivotal for the social functioning of adolescents with ASD. Given that conversation is a ubiquitous part of our social interaction in the world, it is necessary for the everyday academic, interpersonal, and professional interactions that adolescents and young adults with ASD may encounter (Custer et al., 2021; Detar & Vernon, 2020; Koegel et al., 1992; Sng et al., 2018). Delayed or limited conversational skills may reduce engagement in spontaneous skill demonstrations, thereby hindering their ability to receive valuable information from the social environment and adapt their behavior accordingly (Hume et al., 2009; Lee et al., 2007; Sng et al., (2014). Previous researchers have reported that individuals with ASD make fewer social verbal and nonverbal initiations than their typically developing counterparts (Doggett et al., 2013; Sng et al., 2018). Therefore, interventions targeting delayed or limited conversational skills include prompting, support, and feedback from external agents and stimuli (Hume et al., 2009; Lee et al., 2007).

The extant literature on social communication of individuals with ASD suggests two germane findings. First, conversational challenges and deficits that are present at a young age persist into adolescence and young adulthood (Bross et al., 2022; Custer et al., 2021; Detar & Vernon, 2020; State & Kern, 2012). Second, the body of literature on interventions targeting conversation behaviors in young adulthood is rather limited and there is a clear need to expand the empirical knowledge base to the adolescence and older age range (Chezan et al., 2020; Custer et al., 2021; McKeithan & Sabornie, 2020). Addressing the existing gap by identifying effective interventions is imperative for meeting the conversational needs of adolescents and young adults and expanding the limited empirical knowledge base (Chezan et al., 2020; Ledford et al., 2018; McKeithan & Sabornie, 2020). This challenge is especially critical given the increasingly complex social environments that adolescents with ASD encounter across a variety of social and academic circumstances, expectations, and demands (Bross et al., 2022; Detar & Vernon, 2020). Independent functioning becomes vital as individuals with ASD are expected to engage appropriately in employment, daily living, and relationships (Hume et al., 2009).

The substantial challenges of maintaining appropriate conversational skills are unlikely to ameliorate incidentally, without targeted intervention. Hume et al. (2009) discussed self-monitoring as an advantageous intervention for supporting the conversational skills of older individuals with ASD while facilitating independence and minimizing adult support. Self-monitoring is an intervention procedure whereby individuals with ASD are taught to discriminate their behavior and to record its occurrence or nonoccurrence. When individuals self-monitor conversational skills they are taught to attend to selected aspects of the conversational exchange that they may ordinarily overlook (Bouck et al., 2014; Hume et al., 2009; King et al., 2014). The intervention may present external signals that prompt the self-recording of behavior, such as watching visual prompts or listening to audio cues (King et al., 2014). Self-monitoring has been suggested as particularly appropriate for social communication interventions because monitoring can assist in attending to pertinent cues in the constantly changing social environment during a conversational exchange and in adapting behavior accordingly (Paul et al., 2009; Sng et al., 2014); also, self-monitoring can potentially increase independence in the conversational exchange and contribute to self-determination (Bouck et al., 2014; Lee et al., 2007; Randall et al., 2022).

Research on self-monitoring interventions to address conversational skills in individuals with ASD has produced promising outcomes. Bouck et al. (2014) used a low-tech (i.e., paper and pencil) and high-tech (i.e., iPad) self-monitoring checklist to examine task completion of three adolescents (age 13–15) with ASD. Results showed increased task independence and reduced prompting and these findings maintained for 14 weeks (Bouck et al., 2014). Randall et al. (2022) used a self-monitoring checklist within a self-determination choice-making curriculum for six adults (age 20–23) with intellectual or developmental disabilities. Participants’ choice-making improved when self-monitoring was applied. While self-monitoring is often categorized as a consequence treatment component (Ledford et al., 2018), researchers (e.g., Hume et al., 2009; King et al., 2014) have also suggested that it may be more advantageous when supplemented by an antecedent visual support such as video-based models (Hume et al., 2009; King et al., 2014; Ledford et al., 2018).

Video-based models capitalize on individuals with ASD responsiveness to visual stimuli to support the conversational skill (Ayres & Langone, 2005). Ledford et al. (2018) explain that the video-based model involves an antecedent manipulation and serves as a visual prompt to support the target conversational response. The video-based model presents vivid depictions of the natural environment and primes the individual with ASD to discriminate and predict the targeted future social interaction or the expected conversational response and essentially makes the expected response more salient (Ayres & Langone, 2005). Additionally, when the individual with ASD receives the opportunity to observe the desired conversational behavior and the consequences that follow, a target response is more likely to occur (Ayres & Langone, 2005; Hume et al., 2009; King et al., 2014; Sng et al., 2014).

The video-based model is suitable for supporting existing delayed or limited conversational skills as it explicitly shows an individual how to behave in a given social situation, highlights pertinent discriminative stimuli of performance such as use of words and volume, and allows as many replays of the demonstration as necessary (Sng et al., 2014). The visual and auditory information enhances the individual’s understanding of the surrounding physical and social environment, and other rather abstract concepts such as the rate of conversation and the sequence of interactions (Rutherford et al., 2020). Ayres and Langone (2005) also suggested that for individuals who have already acquired appropriate conversational skills, the video-based model can function to enhance their awareness to their conversational response and its adaptation to the social environment. Rutherford et al. (2020) nonetheless discern that individuals will need personal motivation to engage with the conversational skill. Thus, programming a consequence treatment component, such as self-monitoring, in addition to visual support, is essential.

Consistent with the findings in Ledford et al.‘s (2018) comprehensive literature review, which showed that video-based interventions were only occasionally used, we were able to locate only two examples that used self-monitoring combined with visual supports (although not video-based model) to address the conversational skills of adolescents and young adults. Bross et al. (2022) employed a smartphone digital self-monitoring application and a paper graphic organizer to enable participants (*n* = 3, age 21–26) with ASD to list facts about their conversational partners and potential conversational topics and questions to ask. The participants were engaged in a natural conversation that was based on their preferences with a conversational partner for 10 min. In the self-monitoring condition, the participants selected a goal for the number of questions to ask, and the digital app delivered an auditory and visual prompt at 1-min intervals to self-monitor question asking and to select “yes” or “no” in the app. The visual-support condition included the graphic organizer in addition to the self-monitoring app. Question asking was improved for all participants when self-monitoring was employed alone, while the visual-support effects varied across the participants.

In another study, State and Kern (2012) evaluated and compared the effectiveness of self-monitoring and video feedback on the social interactions of an adolescent (age 14) with ASD requiring minimal support. The participant had challenges in social interactions with others and demonstrated inappropriate behaviors in social situations such as repetitive questions and talk and inappropriate noises. The participant engaged in an interactive game activity that required turn-taking with his mom or a friend. He used a self-monitoring sheet to monitor his social interaction and record whether his response was appropriate via a yes/no choice response. In the self-monitoring condition, the participant received a tactile prompt every 15 s. In the video feedback condition, he performed self-monitoring of the videotaped game in the same manner. Points were awarded for monitoring appropriate social responses and for responses that matched with the facilitator’s response. At the end of each session the participant exchanged the points he earned for computer time. Both interventions increased appropriate social interactions, with reductions in inappropriate interactions and noises being greater in the self-monitoring conditions (State & Kern, 2012). The two studies, though limited in participants, show positive outcomes for self-monitoring. However, when combined with visual supports, the results are inconsistent despite the promising potential of these supports for encouraging appropriate conversational responses (Ledford et al., 2018). Furthermore, the literature lacks valuable information on the use of self-monitoring as a primary consequence treatment component, especially when supplemented with video-based models as an antecedent treatment component to promote the conversational behavior of adolescents with ASD.

The current investigation aimed to extend the available information on interventions addressing the conversational needs of adolescents with ASD by examining the effectiveness and acceptability of a self-monitoring intervention supplemented by a video-based model. The primary research question was: What is the effect of self-monitoring, supplemented by a video-based model on the conversational skills of adolescent students with ASD? The second question was: What is the acceptability of the intervention among the participating adolescents with ASD?

## Method

### Participants

Three students, enrolled in a special education school for individuals with ASD who require low to moderate support, participated in this study. They attended a multi-age classroom for students in Grades 10 through 12. The homeroom teacher recruited participants who met the following eligibility criteria: (a) a formal diagnosis of ASD and an eligibility for placement in a special education school for ASD determined by a formal ministry of education committee; (b) be at the age of 13–21; (c) demonstrate delayed or limited verbal social communication skills in one or more of the skills of taking turns, asking and responding questions, and making on-topic comments; and (d) attend school regularly. Two expert special education teachers who specialize in students with ASD and have experience teaching the participants validated the students’ selection for the study and confirmed that they met the eligibility criterion of delayed or limited verbal social communication skills. One of these experts was the experimenter (i.e., second author of this study), who was a special educator in the school and an applied behavior analysis graduate student. She had previously taught the participants for 1–2 years. The research was approved by the university research committees and the ministry of education, and participants and their guardians provided informed consent to participate in the study.

### Oscar

Oscar was an 18-year-old male student with ASD who lived with his mother. The diagnostic assessments of ASD included the Autism Diagnostic Observation Schedule, Second Edition (ADOS–2), which indicated Level 1, requiring support; The Childhood Autism Spectrum Test (CAST), which yielded a score of 22; and the Wechsler Intelligence Scale for Children, Fourth Edition (WISC–VI), which documented performance below age level. The evaluation identified persistent deficits in social communication and social interaction, manifesting as difficulties in managing conversation and reciprocal interaction. Oscar’s individualized education plan included speech therapy, occupational therapy, and art therapy in school, and he also had a weekly meeting with a psychologist in the community. Oscar’s reading was comparable to that of a late elementary student. While he could read short, basic texts with relative fluency and accuracy, his reading comprehension was significantly lacking. His writing skills were at an early elementary level. Though he could hold a pencil correctly and write simple sentences, he often avoided writing tasks, which hindered the development of his writing skills. Oscar struggled in social communication with friends and adults. Despite having verbal abilities and proper vocabulary, he often sought attention by physically clinging to others. His verbal social communication was usually brief and swift using short sentences. He rarely commented on others’ statements, and his responses to questions were typically abbreviated one-word answers such as “yes” or “no” and “I don’t know.” Additionally, he seldom asked questions of others.

### Rachel

Rachel was an 18-year-old female student who lived in a group home for adolescents and adults with mild to moderate ASD during weekdays and with her parents on weekends. Her primary diagnosis was ASD, comorbid with attention-deficit-hyperactivity disorder, behavioral and emotional difficulties, intellectual disability, and sensory processing disorder. The diagnostic assessments for ASD included the ADOS–2, which indicated Level 2, requiring substantial support, with a social affect score of 8, a restricted and repetitive behavior score of 4, and a total test score of 12; The Autism Diagnostic Interview-Revised (ADI–R) yielded a score of 20 in the Quality of Social Interactions section, and a score of 7 in both the Communication section and the Restricted and Repetitive Behaviors section. The Adaptive Behavior Assessment System, Second Edition (ABAS–II) documented overall adaptive performance below age level across the three domains, with score of 49 in the Conceptual domain, 40 in the Practical domain, and 55 in the Social domain. Her individualized education plan included speech therapy, occupational therapy and individual drama therapy in school and she also participated in group activities like sports provided by the group home where she lived. Rachel’s reading skills were comparable to those of a late elementary student. She could read page-long basic texts with relative fluency, accuracy and comprehension, especially when the texts were of personal interest. However, her writing skills were more comparable to those of an early elementary student. Her handwriting was rudimentary, and she required significant support to write complete sentences, relying heavily on copying. Without assistance, her written responses were often limited to one-word answers. Similarly, her oral responses ranged from one-word answers to a full sentence when asked questions. Rachel could initiate short, basic conversations with staff and friends, but only on topics that interested her. She often used single words to compliment others, such as saying “enjoy” when someone wore a new clothing item. She also responded to others and posed questions exclusively on topics of personal interest, such as saying “She is my favorite singer.” Rachel faced challenges with discourse on topics unrelated to her interests, including difficulties with turn-taking, pausing her talk to allow others to speak, and using appropriate conversational volume (i.e., she tended to speak loudly). Throughout the school day, Rachel used a behavioral contract which detailed behavioral expectations, required tasks, and her selected reinforcers for meeting the requirements.

### Paul

Paul was a 16-year-old male student with ASD who split his time between living with his mother and stepfather and staying with his biological father. He had attended a special education classroom in elementary school and a special education school specializing in ASD since the age of 12. Although he was verbal, his individualized education plan documented substantial speech deficits including fast-paced and unclear speech, and challenges maintaining conversation topics and taking turns during conversations. The individualized education plan included speech therapy, occupational therapy, and art therapy. Academically, his reading and writing skills were at an early elementary level. As a slow reader, Paul required considerable support such as having texts and questions read to him and being guided toward answers. He occasionally made errors in reading and writing, and his comprehension was notably low. Despite strong copying and drawing skills, he could write independently only in single words, typically those of personal interest. His reading and writing performance improved in 1:1 learning when he was engaged, compared to group learning contexts. In terms of communication, Paul, who was bilingual, used complex sentences and ranging intonation in his verbal communication. He enjoyed socially communicating with staff, although he mostly preferred to be the speaker rather than engaging in a balanced conversation. His speech tended to be swift, monotonous, and continuous, often repeating the same messages without pausing to listen or check in with the listener. He also had a tendency to persist in talking even when the conversation partner was busy or unavailable. He often asked questions related to his own interests (e.g., “Which instructional aide will be with me?“), and rarely inquired about or responded to comments from others, showing limited interest in the conversation partner.

### Setting and Materials

The study took place in a special education school for students with ASD requiring low to moderate support in a large city in a middle eastern country. Two sessions of the first baseline phase were conducted in a small office approximately 10 square meters with conventional office furniture (e.g., two chairs, table, computer, and file cabinets). The rest of the study sessions were conducted in an unoccupied empty classroom approximately 18 square meters, equipped with a round table and three chairs. Also present in the rooms were two laptops for data collection and video viewing, a Galaxy Note 20 Ultra smartphone camcorder to record all sessions, self-monitoring sheets (in the intervention sessions only) and writing instruments. A flexible universal phone holder was used to place the camera on a table adjacent to the conversation location, approximately 100 cm from the participant. The camera was used from the first baseline session to desensitize participants’ reactivity.

Two researchers (i.e., the experimenter and a speech therapist specializing in autism who was also an applied behavior analysis graduate student) were the adult video models. Based on the adult-model interventions reviewed in Sng et al. (2014), they created four scripted visual support videos, modeling appropriate and inappropriate conversational response examples (two of each) of 28–44 s each. An example is shown in Appendix A. The researchers preplanned conversational text for each video along with staging comments so that the two example videos explicitly showed appropriate turn taking, asking and answering a question on topic and commenting on topic, and the inappropriate examples explicitly showed talking over one another, commenting topics that were of no shared interest, and not responding to questions when asked. Video models’ conversation topics specifically targeted topics of interest to the participants such as going on a school field trip. The researchers sat side-by-side, approximately 50 cm apart, in an angle such that they were facing each other but also facing the camera.

The researchers used an Asus ZenBook 14 with a 16.9 mm screen to view all videos (i.e., video models and all conversational sessions of the study) in the video-modeling intervention phase and for data coding of all study sessions. The participants used a size A4 self-monitoring sheet to monitor appropriate and inappropriate conversational responses, as shown in Appendix B. Four prompting questions were presented in each row for self-monitoring: Did I take turns? Did I respond on topic to a question asked? Did I ask a question on topic? Did I make a comment on topic? Below each prompting question were two symbols, a green and a red thumbs-up. Participants recorded their behavior by circling the green symbol for “yes” and the red one for “no.”

Lastly, the researchers developed a list of 12 age-appropriate conversation categories (e.g., music and arts, trips, holidays, and food) that they presented to the participants to choose from prior to each conversational session in the intervention phase. For each category the researchers developed a few leading questions (e.g., “Who is your favorite singer?“) to facilitate the conversation for their own use in case the participant needed prompting. These questions were not available to the participants.

### Dependent Variable and Measurement

Based on definitions used by State and Kern (2012), the dependent variable, appropriate conversational behavior, was defined as a sequence of a turn-taking response (i.e., waiting quietly until the speaker finished talking), followed by a verbal utterance that included (a) making a statement or responding on topic, and/or (b) asking a contextually appropriate “wh”- question. The definition was extended for Oscar to commenting, asking, or responding in full sentences.

The researchers collected direct observation data for 6 weeks from the recorded 10-min conversation sessions using an event recording system. The experimenter provided a casual natural conversational opportunity which was a discriminative stimulus for a conversational response. The casual opportunity was an explicit leading question or a comment (“What equipment are you planning on taking to the field trip?“) together with respective intonation, accentuation, and/or expectant facial expression with time delay. The minimal number of casual opportunities for a conversational response were eight (Oscar and Paul) to 13 (Rachel). For a conversational behavior to be coded as appropriate, a turn-taking response had to be demonstrated along with a verbal utterance (i.e., a statement or response on topic, and/or a contextually appropriate “wh”- question). If turn-taking did not occur, or if the subsequent verbal utterance did not occur or was inappropriate (e.g., a statement that was off topic), the conversational behavior was coded as inappropriate. Appropriate conversational behavior was presented as the percentage of response by dividing the sum of the number of appropriate conversational behaviors by the total number of casual conversational opportunities in a conversation session provided and multiplying by 100.

### Study Design and Procedures

A withdrawal ABAB design was conducted to evaluate the effects of self-monitoring supplemented by video-based modeling on the participants’ conversational behavior. The ABAB design can provide a powerful demonstration of causality between the independent and the dependent variable by potentially showing three demonstrations of basic effect (i.e., A1-B1, B1-A2, and A2-B2) and replicated with three participants (Gast et al., 2018). It is suitable for this study as both self-monitoring and visual support in the form of a video model can be easily discontinued (Gast et al., 2018), and it is reasonable to use with these supporting interventions as illustrated in the study by Zimmerman et al. (2017). Lastly, an ABAB design is suitable to behaviors suspected to be under the control of current environmental variables (Gast et al., 2018). In our case, the participants limited conversational behavior that was emitted appropriately only infrequently, has presumably been under environmental contingencies insufficient to produce it at socially-acceptable levels and therefore should be supported (Hume et al., 2009; Lee et al., 2007). The study lasted a total of 8 weeks, and consisted of four baseline sessions, six intervention sessions, five baseline sessions, and four intervention sessions.

### Baseline Phase

During the baseline phase, the experimenter held a 10-min conversation with each participant in a separate room outside the classroom. Baseline conversation sessions occurred 2–3 times a day. Participants were not provided with any instructions on the rules of the conversation or the expected conduct during the conversation, their behavior was not praised, and they were not given feedback for their performance. In each conversational session the experimenter and the participant sat 50 centimeters apart and faced each other. The experimenter greeted the participant at the beginning of the session and engaged in a conversation with them on a topic they expressed their interest. If the participant did not raise a topic of interest, the experimenter asked a prompting question: “What are you interested in talking about today?” and provided support as needed to choose a topic. Then she held a casually flowing conversation.

### Intervention

Intervention conversation sessions occurred 2–3 times a day and were conducted by the experimenter in a separate room outside the classroom. All conversation sessions consisted of a 10-min conversation with self-monitoring. The first conversation session of the day (i.e., sessions 5, 7, 9, 16, 17) was preceded by a 10-min viewing of the video-based model, thereby supplementing the self-monitoring procedure with a video-based model in 50% of the intervention. Sitting arrangement was similar to baseline and a self-monitoring sheet and a pencil were available on the table. When the video-based support was applied, the participant viewed two premade videos, one for inappropriate conversational behavior and one for appropriate behavior. Each video was viewed twice to enhance the visual support. The first viewing was nonstop, and the second viewing was paused 3–4 times, following a demonstration of a chief appropriate (e.g., when one responded on-topic to a question) or inappropriate behavior (e.g., when one interrupted the words of the other). In these pauses, the experimenter asked the participant to use the self-monitoring sheet to assess the conversational behavior in the observed video. She mediated and supported learning by asking guiding questions (e.g., “Can you see that she is still talking?“), directing the participant’s attention to the primary discriminative auditory or visual stimulus (e.g., “Look at her lips. Can you see they are still moving and can you hear that she is still talking?“) and replaying the video as needed for repeated observations. The participant received vocal praise and feedback for completing the monitoring.

The conversation session was held similar to baseline conditions with the addition of self-monitoring. The experimenter asked the participant to select a topic for conversation from the topic categories list and reminded the participant of expected conversational behavior and that she would pause the conversation periodically and ask the participant to self-monitor their conversational behavior similar to the practice with the video model. The experimenter briefly paused the conversation at a two-per-minute rate, and at naturally appropriate moments, to verbally prompt the participant to self-monitor their conversational behavior. In each pause, the participant was prompted to self-monitor the turn-taking response and another relevant conversational response (i.e., on-topic response, comment, or question) so that in most trials two responses were marked for the trial. At the end of each trial the experimenter provided affirmation for correct monitoring or corrective feedback for self-monitoring errors. At the end of the session the experimenter reviewed the participant’s performance on the self-monitoring sheet and provided praise and feedback for their conversational and self-monitoring behavior (e.g., “Nice job keeping your turns” or “Look at how many green thumbs-up you have earned!“).

### Interobserver Agreement (IOA)

The two researchers had 45 h of graduate training and experience in direct observation data collection methods and implemented the following steps to ensure the reliability of the data collection procedures. They developed the operational definition of conversational behavior, the video-based model scripts (i.e., appropriate and inappropriate conversational behavior) and the data collection form together. Next, they piloted the data collection system together using the video-model scripts and achieved 100% agreement. At the conclusion of the study, the researchers met together via zoom technology to complete a trial-by-trial interobserver agreement (IOA) reliability on a 30% random sample of the recorded study sessions, evenly distributed across baseline and intervention conditions. They observed each video together and filled the data collection form independently. If any discrepancies emerged, the researchers replayed the video as necessary, identified the gaps and held a discussion to resolve the conflict if possible.

To calculate a trial-by-trial agreement, the researchers compared the codes for each trial and noted agreement or disagreement. Next, they divided the sum of agreements by the total number of opportunities for agreements and multiplied by 100. Mean IOA data for appropriate conversational behavior during the four research phases (i.e., baseline, intervention, baseline, intervention, respectively) were as follows: 97% (no range), 94% (range: 88–100), 100% (no range), and 100% (no range) for Paul; 96% (no range), 95% (range: 90–100), 98% (range: 90–100), and 100% (no range) for Oscar; and 100% (no range), 99% (range: 97–100), 100 (range: 99–100), and 99% (no range) for Rachel.

### Fidelity of Implementation

The researchers followed recommendations by Ledford and Gast (2014) and created a treatment fidelity checklist consisting of three baseline items and 10 intervention procedures to assess treatment fidelity. The researchers assessed fidelity independently and simultaneously in the same manner as IOA was assessed. Fidelity was assessed for 94.7% of Oscar’s and Rachel’s sessions, and for 84.2% of Paul’s sessions. Average baseline fidelity for all participants ranged from 79 to 89% and average intervention fidelity ranged from 96 to 98%. Fidelity data are displayed in Table 1. Notably, moderate to low fidelity was registered for the item related to selecting a conversation topic in baseline. This inconsistent implementation was due to the experimenter’s response to extraneous events (e.g., a colleague walked into the room when the session began), which caused a drift from the baseline implementation protocol in the initial sessions. The experimenter engaged in self- and peer feedback at the end of the initial baseline and revisited the protocols to improve implementation adherence.

**Table 1:** Treatment fidelity items and level of adherence by the experimenter

	Oscar	Rachel	Paul
Prompt the participant to choose a topic of conversation	40%	67%	57%
Self-monitoring is not conducted	100%	100%	w100%
Video-based model support is not displayed	100%	100%	100%
**Average baseline fidelity**	**79%**	**89%**	**86%**
Define and explain the rules of the conversation	100%	100%	100%
View video-based modeling examples	100%	100%	100%
The participant monitors the behavior in the videos	100%	100%	100%
Support the use of the monitoring sheet as needed during the video model viewing	100%	100%	100%
Allow the participant to choose the topic of conversation	100%	100%	100%
Begin the conversation by commenting or asking a question on the selected topic	100%	100%	100%
The participant self-monitors at the end of a conversation cycle	88.8%	100%	100%
Provide vocal praise on the conversational behavior at the end of the trial	88.8%	100%	100%
Provide corrective feedback for self-monitoring as necessary	100%	100%	100%
Review the conversational behavior performance using the self-monitoring sheet	77.7%	75%	66.6%
**Average intervention fidelity**	**96%**	**98%**	**97%**

The researchers examined IOA on treatment fidelity for baseline and intervention sessions. They observed the video sessions independently and completed the fidelity checklist using a yes/no response items to indicate presence or absence, respectively. A trial-by-trial fidelity IOA was assessed on 94.7% of Oscar’s and Rachel’s sessions, and on 84.2% of Paul’s sessions, and was calculated as explained above. Average IOA for Oscar’s baseline and intervention sessions were 100% and 92%, respectively. Average IOA for Rachel’s baseline and intervention sessions were 92% and 100%, respectively, and for Paul were 100% and 92%, respectively.

### Treatment Acceptability

The researchers evaluated the participants’ acceptance of the intervention’s objectives and procedures after completion of the intervention using a questionnaire containing seven yes/no questions and space to add open comments. An example question was: Were you happy to learn to converse appropriately? The treatment acceptability questionnaire and participants’ responses are presented on Table 2. The researcher administered the questionnaire two weeks after the conclusion of the study by reading aloud each question, and waiting as needed for the participant to circle their chosen answer. Participants completed all answers in approximately 5 min.

**Table 2:** Participants responses to the treatment acceptability questionnaire

Treatment acceptability questionnaire item	Oscar	Rachel	Paul
1. Are you satisfied and happy to learn to converse correctly according to the rules of conversation?	See note	Yes	Yes
2. Is conversing according to the rules of conversation an important action?	Yes	Yes	Yes
3. Do you enjoy following the rules of the conversation during a conversation?	Yes	Yes	Yes
4. Did watching the videos demonstrating a conversation help you?	Yes	Yes	Yes
5. Did it help you measure whether you followed the conversation rules in a self-monitoring form?	Yes	Yes	Yes
6. Do you want to learn more behavior with videos and a self-monitoring measurement form?	Yes	Yes	Yes

## Results

**Fig. 1:**
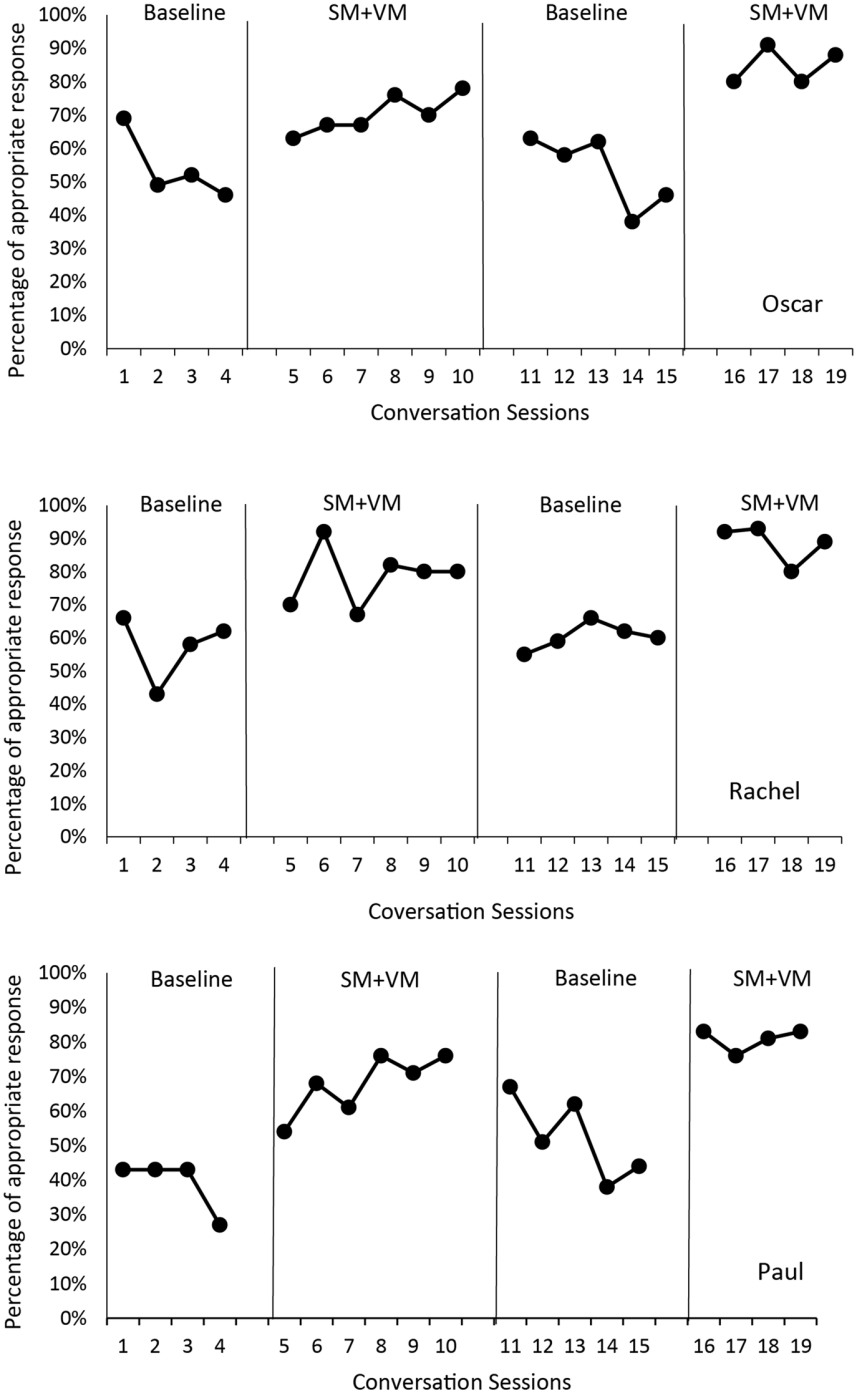
Percentage of appropriate conversational response for Oscar, Rachel, and Paul under baseline and self-monitoring with video-based model (SM + VM)

Results on appropriate conversational behavior for each participant are presented in Fig. 1. We visually inspected the data for changes in data level, trend, stability versus variability, and immediacy of changes with respect to the research conditions.

Oscar’s appropriate conversational behavior demonstrated decelerating trends in both baseline conditions (average 54% and 53% in the first and second baseline phases, respectively). Under the first intervention, Oscar’s appropriate conversational behavior consistently accelerated (average 70%) in the first intervention condition, and increased in level to a fairly stable average of 85% in the second intervention condition. Changes in behavior level from phase to phase were immediate.

Rachel’s appropriate conversational behavior was variable in the first baseline phase averaging 57% (range: 43–66%) and stabilized at the latter part of the first intervention condition, averaging 79% (range: 67–92%). Appropriate conversational behavior decreased in level displaying stable average performance of 60% (range: 55–66%) in the second baseline phase, and increased in level to a relatively stable average of 89% (range: 80–91%) in the second intervention condition. Changes in behavior level from phase to phase were immediate.

Paul’s appropriate conversational behavior in the first baseline phase was relatively stable and showed an average of 39% (range: 27–43%) and consistently decelerated in the second baseline condition, averaging 52% (range: 38–67%). Levels of appropriate conversational behavior immediately increased and displayed accelerating trends in the first and second intervention phases, averaging 68% (range: 54–76%) and 81% (range: 76–83%), respectively.

All participants responded positively to all questions on the treatment acceptability questionnaire with one exception. Paul vocally reported that he was partially satisfied with learning to converse according to the rules of social conversation as he “had to stop and listen to the other person.”

## Discussion

This study’s primary research question was: What is the effect of self-monitoring supplemented by a video-based model on the conversational skills of adolescent students with ASD? Findings indicate that the intervention resulted in improved conversational behavior for all three participants: They asked questions on the selected topic, responded on topic to questions, made comments on topic, and maintained turns in conversations. The improvements were functionally related to the intervention. The second question was: What is the acceptability of the intervention among the participating adolescents with ASD? The treatment acceptability results show the participants agreed that conversational behavior was important and that the intervention assisted in improving their performance. Their agreement suggests the suitability of the intervention’s aim and procedures for adolescents with ASD.

This study extends the available information on effective interventions for addressing the conversational skills of individuals with ASD by specifically examining self-monitoring as a consequence treatment component, supplemented by an antecedent video-based model component. The study also contributes to the relatively limited body of literature on conversation behaviors in young adulthood (Chezan et al., 2020; Custer et al., 2021; McKeithan & Sabornie, 2020). Collectively, this information can guide the evaluation and planning of appropriate interventions designed to improve conversational behaviors of adolescents with ASD.

The improved conversational behavior induced by the self-monitoring treatment supplemented by the video-based model in this study aligns with findings from previous studies, which reported enhancements in question asking (Bross et al., 2022) and appropriate interactions (State & Kern, 2012), gains in choice-making behavior (Randall et al., 2022) and increased independence (Bouck et al., 2014). The consistently positive and compelling outcomes demonstrated across all participants in this study, facilitated by a robust research design, add empirical information to the existing body of literature on self-monitoring combined with an antecedent visual support as an effective treatment to support individuals with delayed or limited communication skills.

The quality and immediacy of the behavioral change under the self-monitoring with the video-based model supplement warrant discussion. Participants in our study exhibited delays or limitations in their existing communication skills, which explains the initially limited to moderate levels of appropriate conversational behavior at baseline. The average improvement in appropriate conversational behavior from baseline to intervention conditions was close to 25% for Oscar and Rachel and 10% for Paul. Rates of appropriate conversational behavior were higher in the second implementation of the intervention than in the first and exceeded an 80% level. Altogether, the findings suggest that the participants’ conversational behavior consistently improved and that quality of performance reached moderate to high levels when participants engaged in self-monitoring supplemented by the video-based model support. Whether the improved level of conversational performance is of clinical importance is a question that should be weighed functionally for individuals with ASD. Moderate or high conversational performance level in this population is determined by its functionality in natural, generalized settings, preferably with the minimal support necessary for the individual. Ledford et al. (2018), however, argue that some support may need to be sustained even after the programmed intervention concludes, to ensure the maintenance of the behavior. Our anecdotal observations at the end of the study illustrated that the participants required the continued use of the supportive intervention to maintain the improved levels of performance and to prevent a potential revert to baseline levels. Both self-monitoring and visual supports, when sustained, are apt to facilitate and maintain improved functioning, even under reduced adult support (Bouck et al., 2014; Hume et al., 2009; Ledford et al., 2018; Lee et al., 2007; Randall et al., 2022).

The immediacy of the behavioral change should also be considered. Notably, the appropriate conversational behavior of all three participants accelerated immediately when the self-monitoring with the video-based supplement was implemented and decelerated when it was withdrawn. The relatively sudden increase possibly illustrates the role of self-monitoring as a motivating consequence component. Presumably, the unchecked boxes on the self-monitoring sheet might have functioned as a conditioned transitive establishing operation that established checked boxes as conditioned reinforcers and occasioned the behavior required to check the boxes. Our presumption should be considered with caution because we have not conducted a component analysis that could have provided more detailed information on the functionality of each intervention component. However, it is important to recognize that motivating individuals with ASD to engage in conversational behavior at their full potential using natural and automatic social cues is often challenging (Doggett et al., 2013). Additionally, we hypothesize that the use of video-based model (though infrequently displayed), increased the likelihood of the desired response, as suggested by other researchers (Ayres & Langone, 2005; King et al., 2014; Sng et al., 2014). Overall, the self-monitoring intervention, supplemented by a video-based model, helped participants manage their conversational behavior during its application. In its absence, however, the control diminished, leading to a gradual regression of the desired behavior to baseline levels. The favorable treatment acceptability ratings obtained from the participants, consistent with previous research (i.e., State & Kern, 2012), may be another indication of the presence of motivation and the suitability of the intervention components for this age group.

Another motivational factor should be considered for its possible influence on the positive outcomes of this research is the selection of a conversation topic by the participant. The participants were allowed to select conversation topics of their interest, which might have enhanced their motivation to engage in the conversations. Nonetheless, from the participants’ behavior we have learned that conversing on favorite topics associated with elevated motivation also invited frequent opportunities to practice challenges such as inhibition of chatty behavior and practicing taking turns and considering the interests of the conversation partner while delaying one’s own. At least one avenue to explore is the effects of self-monitoring supplemented by a video-based model on the level and quality of the conversational behavior given topics selected by the conversational partner and some that are of no particular interest for the participant.

Finally, while the intervention in this study was primarily designed to evaluate the participants’ use of self-monitoring (primed by an antecedent visual support) and its potential to support their conversational behavior, it did not aim to examine maintenance and generalization. Despite the compelling short-term results of this study, the lack of maintenance and generalization data hinders our understanding of the conversational behavior functioning level postintervention thereby limiting our ability to tout the intervention’s potential long-term effects in the establishment of age-appropriate relationships and memberships in social groups. Maintenance and generalization programming are warranted to achieve clinical importance and ecological validity (Gast et al., 2018), and should be targeted in future research on self-monitoring of conversational behavior in adolescents with ASD. Maintenance and generalization could be fostered by a partial removal of intervention components, such as further minimizing the visual support priming, systematically fading of conditioned stimuli, using self-monitoring instruments that can be available across settings, and practicing the behavior across conversational partners.

### Limitations and Future Research Directions

The results of this study are preliminary, and replications are needed to further evaluate self-monitoring and a video-based model intervention when used with adolescents with ASD for social-communication purposes. There are two primary limitations that warrant discussion. First, the findings are limited to conversational episodes held in an analogue training setting, with an adult partner trained in special education, who used explicit discriminative stimuli in the conversational exchange and who supported the conversational exchange. For example, when a participant was hesitant or slow to respond, the adult waited with an expectant and encouraging facial expression. These mediating salient prompts may not be present in a social conversation with natural partners in school or community settings where they may even be considered socially unacceptable or rather stigmatizing. Future research should program the fading of salient conversational discriminative stimuli to levels at which they would naturally occur with peer conversational partners or other people in the community. Given that engagement with diverse conversational partners is advantageous (Bross et al., 2022), we suggest extending the current study by programming a mixture of conversational situations with peers and with an adult partner and also targeting unprompted naturally-occurring conversational behavior. The possible benefits could be the additive conversational behavior in the presence of more and less salient conversational discriminative stimuli, decreased reliance on adult prompts, and increased probability for generalization.

The second limitation concerns the dependent variable measured in this study. Although we used a relatively wide-ranging definition of conversational behavior consisting of four conversational responses, this study is still limited to aspects of topic management and reciprocity of turn taking. We did not examine other complex conversational responses related to information management such as commenting or providing appropriate amounts of information, attention and sensitivity to the listener’s verbal and nonverbal cues for turn-exchange or topic shift, or properly persevering in a conversation on topic that is not of personal interest. Future research may consider expanding the investigation to additional responses based on the needs of the participating individuals with ASD and could also incorporate additional instruments for assessing the improvement of the conversational behavior, such as the Clinical Global Impression Scale (Toolan et al., 2022). Given that this study is the first to use self-monitoring with a video-based model supplement with adolescents with ASD, additional replication studies with adolescents and adults are needed to validate the current findings.

### Implications for Practice

A primary implication of the current study is the relative ease of programming and implementing the self-monitoring and a video model support procedure. The self-monitoring sheet was convenient to use and not aversive or stigmatizing. It can be easily created and modified to meet the conversational limitations or delays of individuals with ASD. For example, the pictorial and written prompts of the self-monitoring sheet can be changed to focus on responding in full sentences as was the focus for Oscar, or pausing and allowing the listener to respond, which could have hypothetically benefited Rachel’s conversational repertoire.

The conversational video-based models can also be easily prepared and produced due to the enhanced convenience and accessibility of technological recording devices. Videos of 45–60 s are appropriate for adolescents with ASD and do not require increased response effort from those who prepare them, nor from the adolescents who are asked to observe them for practice. They can be used once a day as executed in the current study, on a leaner schedule if the individual requires minimal support, or specifically before target functions such as an upcoming friendly meeting.

## Conclusion

This study successfully programmed self-monitoring supplemented by a video-based model as an intervention designed to meet the conversational needs of three adolescents with ASD. Improving the conversational skills of adolescents and young adults is essential for their independent functional engagement in various activities in adolescence and adulthood such as employment and relationships. Through this research we aspired to use a promising intervention procedure that could potentially meet their needs as they approach the transition into society as independent adults. This study’s findings were positive and so was the overall satisfaction of the participants with the intervention.

## Electronic supplementary material

Below is the link to the electronic supplementary material.

Supplementary Material 1

Supplementary Material 2
