# Supplementary material for: Supporting the Conversational Behavior of Adolescents with Autism Spectrum Disorders with Self-Monitoring and a Video-Based Supplement
Source: J Autism Dev Disord. 2024 Sep 13;56(1):13–25. doi: 10.1007/s10803-024-06548-3 (PMC12860758; doi:10.1007/s10803-024-06548-3)
Supplement: Supplementary file 1 — Supplementary Material 1 [file 10803_2024_6548_MOESM1_ESM.docx]

**Appendix A**

**An Example of Scripts for Appropriate and Inappropriate Conversational Behavior**

| Appropriate | Inappropriate |
| --- | --- |
| Conversation topic: Eating ice-cream | |
| Partner A: "Hey, Isla, how are you?" (Partner A sits facing Isla and looking at her)  Partner B (Isla): "Ok" (maintaining eye contact)  Partner A: "Do you know what happened to me yesterday?" (taking turns)  Partner B: "What happened yesterday? Do tell!" (Asking a question on topic)  Partner A: "I went to the shopping center and bought ice cream. Do you know which flavor I chose?"  Partner B: "Which flavor?" (responding on topic and asking a question on topic)  Partner A: "White chocolate and strawberry cream" (Answering a question on topic)  Partner B: "I would love eating ice cream with you sometime" (responding on-topic)  Partner A: "Let's do it next time we go together to the shopping center" (responding on topic)  Partner B: "Ok, I have to go to class." | Partner A: "Hey, Isla, how are you?" (Partner A sits facing Isla and looking at her)  Partner B (Isla): "Ok" (her head is tilted down, and she does not look at partner A)  Partner A: "Do you know what happened to me yesterday?"  Partner B: "What a boring class that finally ended. I almost fell asleep." (The two speak at the same time; do not take turns in the conversation)  Partner A: "I went to the shopping center and bought ice cream. Do you know which flavor I chose?"  Partner B: "The teacher gave us a worksheet which I didn't finish, so now I have homework, ugh! (No question asked, no response on topic)  Partner A: "What class was that?" (Question asking)  Partner B: "I'm really hungry." (commenting off topic, no response to the question asked)  Partner B: "I have to go to class" (Get up and go) |
