# Supplementary material for: Supporting the Conversational Behavior of Adolescents with Autism Spectrum Disorders with Self-Monitoring and a Video-Based Supplement
Source: J Autism Dev Disord. 2024 Sep 13;56(1):13–25. doi: 10.1007/s10803-024-06548-3 (PMC12860758; doi:10.1007/s10803-024-06548-3)
Supplement: Supplementary file 2 — Supplementary Material 2 [file 10803_2024_6548_MOESM2_ESM.docx]

**Appendix B**

**An Example of a Self-Monitoring Sheet**

| 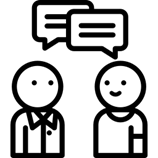 | Did I take turns?  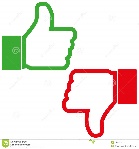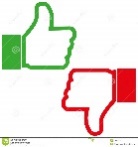 | Did I respond on topic to a question asked?  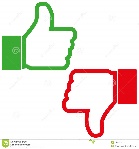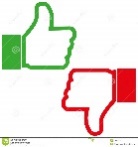 | 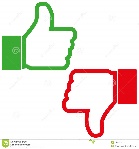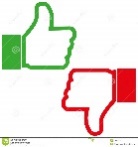Did I ask a question on topic? | Did I make a comment on topic?  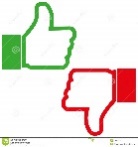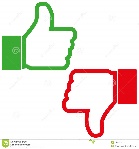 |
| --- | --- | --- | --- | --- |
| 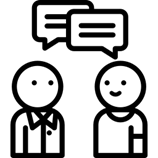 | 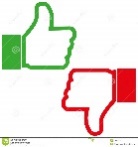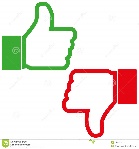Did I take turns? | Did I respond on topic to a question asked?  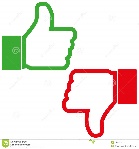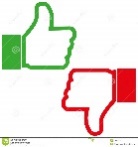 | 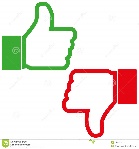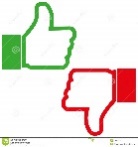Did I ask a question on topic? | Did I make a comment on topic?  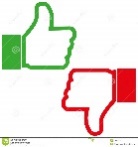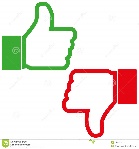 |
| 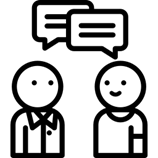 | 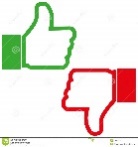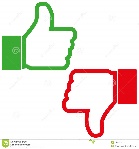Did I take turns? | Did I respond on topic to a question asked?  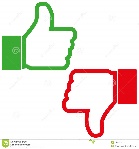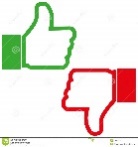 | 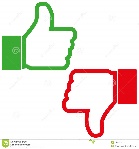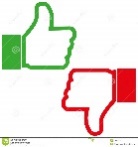Did I ask a question on topic? | Did I make a comment on topic?  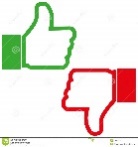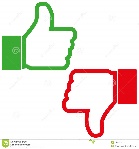 |
| 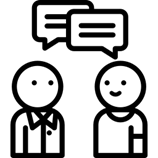 | 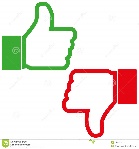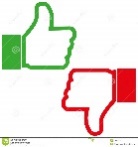Did I take turns? | Did I respond on topic to a question asked?  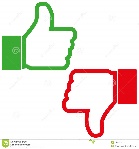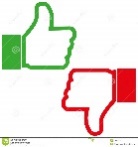 | 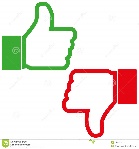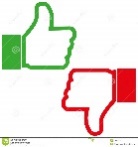Did I ask a question on topic? | Did I make a comment on topic?  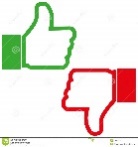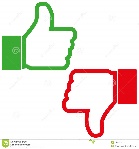 |
| 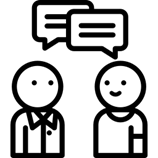 | 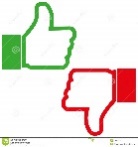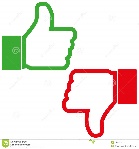Did I take turns? | Did I respond on topic to a question asked?  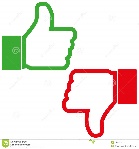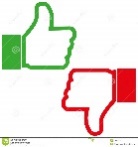 | 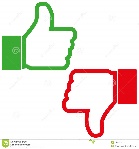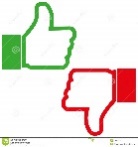Did I ask a question on topic? | Did I make a comment on topic?  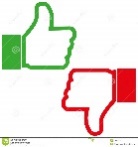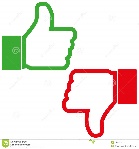 |
